# Surveillance Service of Yellow Fever in Non-Human Primates in the Federal District, Brazil, 2008-2022

**DOI:** 10.1590/0037-8682-0225-2025

**Published:** 2026-02-02

**Authors:** Gabriela Rodrigues de Toledo Costa, Pedro Henrique de Oliveira Passos, Davi Emanuel Ribeiro de Sousa, Isabel Luana de Macêdo, Daniel Garkauskas Ramos, Karina Ribeiro Leite Jardim Cavalcante, Alessandro Pecego Martins Romano, Arnaldo Jorge Martins, Livia Medeiros Neves Casseb, Livia Caricio Martins, Nicole Lynn Gottdenker, Eduardo Mauricio Mendes de Lima, Cristiano Barros de Melo, Márcio Botelho de Castro

**Affiliations:** 1 Universidade de Brasília, Programa de Pós-Graduação em Ciências Animais, Campus Darcy Ribeiro, Brasília, DF, Brasil.; 2 Secretaria de Saúde do Distrito Federal, Gerência de Vigilância Ambiental de Zoonoses, Brasília, DF, Brasil.; 3 Ministério da Saúde, Secretaria de Vigilância Sanitária, Brasília, DF, Brasil.; 4 Universidade de Brasília, Hospital Veterinário Universitário, Laboratório de Patologia e Perícia Veterinária, Brasília, DF, Brasil.; 5 Instituto Evandro Chagas, Levilândia, PA, Brasil.; 6 University of Georgia, Department of Veterinary Pathology, Georgia, Athens, United States of America.

**Keywords:** Marmoset, Callithrix, Outbreak, Disease, Death, Investigation

## Abstract

**Background:**

Surveillance of non-human primate (NHP) deaths is vital for the early detection of yellow fever (YF) and prevention of its spread to the human population. This study assessed the YF surveillance system for NHPs in the Brazilian Federal District (FD) from 2008 to 2022.

**Methods:**

A retrospective analysis of the aggregated data from 15 years of outbreak surveillance involving NHP deaths was conducted. The analyzed variables included spatiotemporal distribution, species, sex, age, sample collection, cause of death, and YF test results.

**Results:**

In total, 1,175 outbreaks involving 1,353 NHP deaths were recorded, averaging 1.35 animals per outbreak, in urban and peri-urban areas. Twenty YF-positive outbreaks were confirmed in 2008, 2015, and 2020, affecting 27 animals, mainly adult *Callithrix* spp., with an overall YF positivity rate of 2%. Surveillance coverage expanded across all administrative regions of FD, with 96.7% of NHP deaths sampled for YF and pathological analysis. Over the last 5 years, the rate of conclusive diagnoses has increased by 60%, with trauma and infectious diseases being the most common causes of death.

**Conclusions:**

The strategic location of FD reinforces the need for ongoing NHP death surveillance as an early warning tool for patients with YF. Continued enhancement of the diagnostic capacity and data integration is essential for strengthening the prevention and control efforts of YF in Brazil.

## INTRODUCTION

Epizootic surveillance in non-human primates (NHPs) plays a key role in Brazil's public health system, primarily by enabling early detection of yellow fever virus (YFV) transmission. As part of the National Program for the Control of Yellow Fever by the Brazilian Ministry of Health (BMH), the detection of YF in primates supports official health surveillance services in making timely decisions to protect human populations, thereby reducing the morbidity and mortality associated with YFV, particularly in areas with active or potential transmission^1^ ^-^ ^3^.

Historically, YF has emerged during different periods, causing outbreaks in free-ranging NHPs primarily in the Amazon region, but has also spread to other parts of Brazil. These outbreaks typically begin in sylvatic environments and may spill over into vulnerable human populations^1^ ^,^ ^4^ ^,^ ^5^. NHPs are competent hosts for YFV and exhibit both fatal and subclinical infections without any pathological findings^1^ ^,^ ^2^ ^,^ ^6^ ^-^ ^9^. Non-human primates serve as important sentinels for YF, providing early warnings that enable public health services to implement preventive measures.

The Federal District (FD) is the smallest unit of the Brazilian Federation and is located in Central Brazil (Midwestern Region), a transitional area between the Amazon and the densely populated Southeastern/Southern Regions. In addition, extensive forest fragments of the Savanna biome (Brazilian Cerrado) are interspersed among densely populated urban areas within the FD, providing suitable habitats for the principal YF vector mosquitoes that are abundant in this region^10^. Therefore, the strategic location of FD makes it crucial to conduct YF surveillance in NHPs to predict the spread of the disease to more populated regions. We evaluated the Surveillance Service of YF in NHPs in the Federal District, Brazil, from 2008 to 2022.

## METHODS

A retrospective study was conducted over 15 years (2008-2022) using an aggregated dataset from the surveillance service of outbreaks of death in non-human primates (NHPs) conducted by the Environmental Zoonosis Surveillance Management, Directorate of Environmental Health Surveillance, Secretariat of Health from the Federal District (GVAZ/SES-FD), Brazil. Primary data were retrieved from the Epizootic Notification Forms of the Notifiable Diseases Information System (SINAN, Brazilian Ministry of Health) and notification spreadsheets of deaths in NHPs from the GVAZ/SES-FD, under the authorization of the Directorate of Environmental Health Surveillance, DIVAL/SES-FD.

GVAZ/SES-FD gathered all NHPs found dead in the Federal District, epidemiological reports of deaths were recorded, and tissue samples were collected during necropsies for histological evaluation, immunohistochemistry, and molecular assays (RT-PCR, available since 2017) for YF detection in the reference laboratories of the Brazilian Ministry of Health (BMH), including the Evandro Chagas Institute (IEC), Adolfo Lutz Institute (ADL), and the Veterinary Pathology and Forensic Laboratory of the University of Brasília (VPFL/UnB). The diagnosis of YF in the BMH reference laboratories includes IHC using alkaline phosphatase with a polymer method^9^ ^,^ ^11^ and RT-PCR^12^.

The data analyzed in this study included the spatiotemporal distribution of outbreaks in NHPs; the number of animals involved in outbreaks; species, sex, and age of the animals; the number of samples collected and sent to the BMH Reference Diagnostic Laboratories; primary causes of death; and laboratory results for YF tests. The number of recorded outbreaks with deaths in NHPs was also grouped and compared at 5-year intervals from 2008 to 2012, 2013 to 2017, and 2018 to 2022. The causes of death in the NHPs were classified as traumatic injuries, infectious/parasitic diseases, others, or inconclusive. 

Statistical, descriptive, and frequency analyses were performed using Excel 365 and GraphPad Prism 8.0. The QGIS 3.28.10 (Firenze) Geographic Information System (GIS) software was used to standardize the geographic data and create maps. Geographic information was obtained from the GeoPortal-DF and Brazilian Institute of Geography and Statistics (IBGE) websites. This study was conducted with permission from the Secretariat of Health of the Federal District (SEI Process 00060-00452543/2023-90).

## RESULTS

**FIGURE 1:**
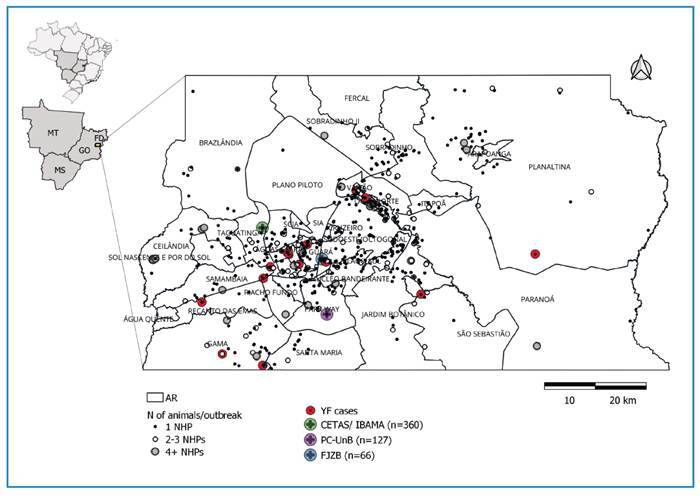
Spatial distribution of outbreaks with the death of NHPs and cases of YF in the Federal District, Brazil, 2008-2022.

Between January 2008 and December 2022, 1,175 outbreaks were recorded, resulting in the deaths of 1,353 NHPs, representing an average of 1.15 dead animals/outbreak. Individual deaths accounted for 91.3% (*n* = 1073/1,175) and multiple animal deaths occurred in 8.7% (*n* = 102/1,175) of the epidemiological investigations (Figure 1). Among the recorded outbreaks, 52.9% (*n* = 622/1,175) were geolocated deaths caused by free-living NHPs. In comparison, 47.1% (*n* = 553/1,175) of the captive or free-living animals were collected by local environmental authorities, for which there was no information on the geographic coordinates of the location of death. These animals received or were kept and died at the Wildlife Screening and Rehabilitation Center (FD), 30.6% (*n* = 360/1,175) (CETAS, Brazilian Institute of Environment and Renewable Natural Resources, IBAMA), Primatology Center UnB, 10.8% (*n* = 127/1,175) (PC-UnB), and Brasília Zoological Garden Foundation FD, 5.6% (*n* = 66/1,175) (FJZB) (Figure 1).

Of the 1,353 animals evaluated in this study, most NHPs were adults of the *Callithrix* genus, followed by *Sapajus*, *Alouatta*, and other genera kept in captivity at FJZB and CP-UnB (Table 1). The Administrative Regions (ARs) of the Federal District (FD) with the highest number of outbreaks in NHPs were Lago Norte (6.9%, *n* = 81/1,175), Lago Sul (5.5%, *n* = 65/1,175), Candangolândia (3.6%, *n* = 43/1,175), Park Way (3.6%, *n* = 43/1175), and Guará (3.5%, *n* = 42/1,175). The ARs with the fewest outbreak notifications in NHPs were Cruzeiro (*n* = 3/1,175), Riacho Fundo II (0.2%, *n* = 3/1175), Sol Nascente (0.2%, *n* = 3/1,175), Riacho Fundo (0.15%, *n* = 2/1,175), SCIA (0.15%, *n* = 2/1,175), Varjão (0.1%, *n* = 1/1,175), and SIA (0.1%, *n* = 1/1,175). All ARs of FD had at least one death in the NHPs recorded, with 46.0% (*n* = 540/1175) of outbreaks occurring in peri-urban areas, followed by 36.0% (*n* = 423/1,175) in urban areas (*p* < 0.01), and 18.0% (*n* =212/1,175) in rural areas (Figure 1).

**TABLE 1:** Genus, gender and age range (%) of NHPs found dead in outbreaks in the Federal District, Brazil, 2008 to 2022.

Genus	Female	Male	Indeterminate	Adult	Juvenile	Indeterminate
*Callithrix* (87.8%, n=1,188)	38.6	44.1	17.3	63.5	24.0	12.5
*Sapajus* (4.4%, n=60)	51.7	43.3	5.0	73.3	25.0	1.7
*Alouatta* (3.6%, n=48)	27.1	35.4	37.5	58.3	10.4	31.3
Others (4.2%, n=57)	52.6	43.9	3.5	73.7	24.6	1.8
**Total (n=1,353)**	**39.4**	**43.8**	**16.9**	**64.2**	**23.6**	**12.3**

**FIGURE 2:**
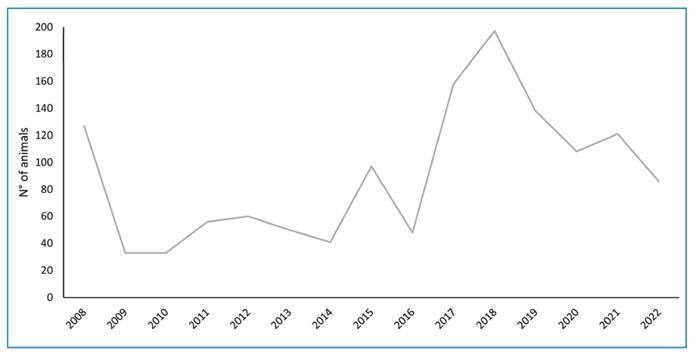
Annual distribution of outbreaks with the death of NHPs in the Federal District, Brazil, 2008-2022.

**FIGURE 3:**
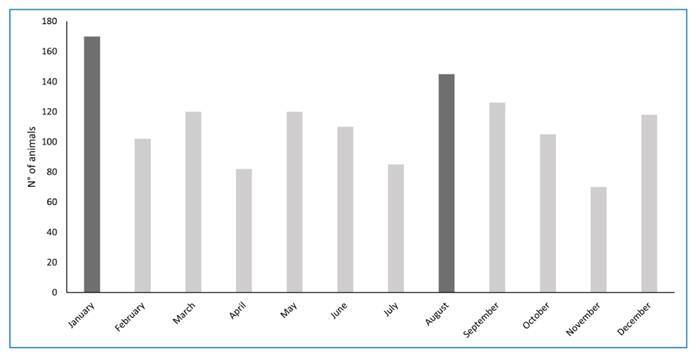
Monthly distribution of outbreaks with the death of NHPs in the Federal District, Brazil, 2008-2022. Months with dark bars showed a high frequency of deaths in the analyzed period.

When analyzing the dataset at 5-year intervals, an increase in outbreaks of deaths in NHPs by GVAZ/SES-FD was noted from 2008 to 2012 (22.2%, *n* = 261/1,175), 2013 to 2017 (30.1%, *n* = 354/1,175), and 2018 to 2022 (47.7%, *n* = 560/1,175) across these periods. The highest number of deaths recorded in NHPs was in 2017 and 2018 (Figure 2). January and August had the highest number of outbreaks, followed by November (Figure 3).

In 96.7% of deaths evaluated in NHPs (*n* = 1,308/1,353), samples were collected for pathological evaluation and referred to the official reference laboratories for YFV detection. Laboratory diagnosis of YF could not be performed in 21.3% (*n* = 288/1,353) of the cases because of inappropriate samples due to advanced decomposition of the carcasses. Diagnosis of causes of death in NHPs was inconclusive in 50.0% (*n* = 654/1,308) of cases (most tested negative for YF, excepting some animals with no tissue sampling due to advanced carcass decomposition), with a marked reduction of around 40 to 42% of inconclusive diagnoses (*p* < 0.01) from 2018 to 2022 (36.9%, *n* = 240/650) compared to 2008-2012 (61.8%, *n* = 165/267) and 2013-2017 (63.7%, *n* = 249/391), respectively. Conclusive diagnoses (50.0%, *n* = 654/1,308) included traumatic injuries (61.3%, *n* = 401/654), infectious diseases (31.2%, *n* = 204/654), and other causes of death (7.5%, *n* = 49/654).

During this study, 20 YF outbreaks were recorded, including 27 animals (1.35 animals detected per outbreak), distributed across three distinct periods: 2008 (positivity of 6.4%, *n* = 3/47), 2015 (positivity of 15.5%, *n* = 14/90), and 2020 (positivity of 9.4%, *n* = 10/106). These outbreaks accounted for 2.5% (*n* = 27/1065) of all NHP deaths in YF. In 2008 and 2015, and one case in 2020, 66.7% (*n* = 18/27) showed hepatic YF-associated lesions, such as midzonal to panlobular hepatic necrosis and estatosis with Councilmann bodies^2^ ^,6,8^, and tested positive for YF via IHC assay, whereas 33.3% (*n* = 9/27) were positive by RT-PCR and showed no liver damage in 2020 (quantification cycles-Cq: 30-37). The genus *Callithrix* was the most affected by YF in FD, representing 85.2% of all cases (*n* = 23/27), with the majority being adults (66.7%, *n* = 18/27).

## DISCUSSION

Surveillance of outbreaks involving NHP deaths in Brazil enables early detection of YFV circulation and informs decisions to prevent YF in vulnerable human populations. Notable examples of effective YF and other outbreak surveillance in NHPs have been reported, primarily in the Southeastern and Southern regions of Brazil^1^ ^,^ ^2^ ^,^ ^3^ ^,^ ^10^, where the implementation of the SISS-GEO application, developed for the geolocation and health surveillance of wild animals, has substantially strengthened disease monitoring, planning of preventive measures, and predictive modeling efforts^13^. However, this information is lacking for Central Brazil.

Over the 15 years of this study, YF surveillance strategies in FD have resulted in the recording of over 1,100 outbreaks, with an annual average of approximately 80 outbreaks, affecting more than 90 non-human primates (NHPs)^14^. The number of NHPs per YF outbreak in FD was lower than that recorded between 2008 and 2009 in São Paulo State (1.6 NHPs/outbreak)^15^, and the national average of 1.8 NHPs detected per outbreak in Brazil from 2007 to 2009^16^. Differences in the genus, density, and distribution of NHP populations, as well as environments and distinct biomes, may explain the differences between FD and other locations.

Despite the surveillance of YF prompting the investigation of NHP outbreaks in FD as an essential component of the National Program for the Prevention of YF, YFV infections accounted for only 2.5% (*n* = 27/1,065) of all dead animals tested for YF and 13.2% of infectious disease cases. These cases were distributed across three distinct periods of YFV transmission (2008, 2015, and 2020) and primarily affected marmosets, with positivity rates ranging from 6.4% to 15.5%. The low positivity rate for YF in NHPs in this study contrasts with most studies conducted on YF surveillance in Northwestern, Southeastern, and Southern Brazil, where positivity rates can reach over 68%, particularly in areas with a high incidence of *Alouatta* ^1^ ^,^ ^2^ ^,^ ^6^ ^,^ ^7^ ^,^ ^17^ ^,^ ^18^. Therefore, surveillance of outbreaks with deaths in NHPs in FD exhibited significant differences from other regions of Brazil, with relatively low positivity rates in NHPs, even during periods of YFV transmission.

*Callithrix* (mainly *C. penicillata*) was the primary genus of free-ranging NHPs involved in FD outbreaks, followed by *Sapajus* (*S. libidinosus*) and *Alouatta* (*A. caraya*). Most affected individuals were adults without a sexual predisposition. These NHP genera are frequently involved in outbreaks in Brazil, although their proportions vary significantly by region^1^ ^,^ ^2^ ^,^ ^3^ ^,^ ^14^ ^,^ ^15^ ^,^ ^18^. The black-tufted marmoset (*C. penicillata*) is particularly well adapted to human-altered environments, thrives in urban and peri-urban areas, and is abundant in the Cerrado biome^19^ ^,^ ^20^, as observed in the FD.

Outbreaks also involved the deaths of capuchins (*S. libidinosus*) and howler monkeys (*A. caraya*), as well as YF cases in these genera, and mainly occurred in environmentally protected and peri-urban areas during the initial years of this study. Over the last 10 years, the marked expansion of urban regions and anthropogenic pressure on natural environments in the FD have likely negatively affected NHP populations. Further studies are needed to investigate the potential causes of the likely population decline of capuchins and howler monkeys, as well as the impact of increased forest fragmentation on FD.

The marked differences in YF positivity in NHPs in FD compared to other locations in Brazil may be related to the higher frequency of black-tufted marmosets and the likely low number of howler monkeys in the region, different environments, and biomes, which may have influenced the dynamics and transmission of YF. Despite the limited number of samples, during the YFV outbreak in 2020, only one in 10 YF-positive *Callithrix* spp. was detected by histopathological evaluation and IHC assay when RT-PCR tests were used concurrently for YF diagnosis in FD. Marmosets have demonstrated low viral loads and/or low rates of death and positivity in YF outbreaks, possibly due to different susceptibilities to YFV infection compared to *Alouatta*, which is considered one of the most susceptible NHP genera to YF in Brazil^1^ ^,^ ^6^ ^,^ ^7^ ^,^ ^2^ ^,^ ^8^. The low positivity rate of YF in this study, primarily determined using immunohistochemistry (IHC) assays, may be attributed to the majority of the tested animals belonging to the *Callithrix* genus, as previously reported^2^ ^,^ ^7^ ^,^ ^8^. In addition, this rate would likely be higher if all samples were tested with highly sensitive molecular assays, such as RT-PCR, which have been used in other YF outbreaks in NHPs, mainly involving *Callithrix* spp. in the Southeast and Northeast regions of Brazil^7^ ^,^ ^17^. 

The FD is located in Midwestern Brazil and forms part of the ecological corridor for the circulation and propagation of YFV. These spatial corridors originate in the Amazon region and extend along a north-south axis, passing through the Midwest region and continuing toward the southeast and South Brazil^5^. The FD serves as a transition zone between the Amazon and the Southeast/South regions, which have the highest human population density in Brazil and variable YF vaccination coverage^18^. Consequently, even with the low positivity rate for YFV, surveillance of outbreaks in NHPs in the Federal District is strategic and crucial because of its geographical location. It plays a key role in raising awareness of new YFV emergence or circulation, and in implementing preventive measures in other Brazilian regions. 

The Health Surveillance Agency of the Federal District (GVAZ/SES-FD) achieved a marked increase of 58%-112% in the records of investigated outbreaks in NHPs from 2018 to 2022 (47.7%, *n* = 560/1175) compared with 2013 to 2017 (30.1%, *n* = 354/1175) and 2008-2012 (22.2%, *n* = 261/1175). January and August had the highest number of NHP deaths. Similar seasonal variations have been observed in other studies in Southeast Brazil, showing differences in the number of NHP deaths across years and months^15^ ^,^ ^18^.

The marked increase in the records of outbreaks involving NHPs in FD over the last 5 years, even during the SARS-CoV-2 pandemic, which negatively impacted disease surveillance globally, can likely be attributed to a public outcry for YF prevention and control. This heightened concern stemmed from one of Brazil's worst YF outbreaks, with over 2,000 cases and hundreds of human deaths in the southeastern Region between 2017 and 2018, particularly in the state of Minas Gerais^4^. This public outcry possibly increased the sensitivity of the population and health surveillance services to NHP deaths, including FD. However, from 2019 to 2022, the number of NHPs collected for YF surveillance in the FD gradually decreased, suggesting a weakening of the local health surveillance service, a decrease in the sensitivity of the population to report deaths in NHPs, and the impact of the COVID-19 pandemic on official health services. This study recorded outbreaks involving NHP deaths, with only a few cases related to YFV infection. Therefore, most cases result from other causes of death in NHP, presenting different epidemiologies and indeterminate seasonality, complicating comparisons with other studies investigating YF outbreaks^15^ ^,^ ^18^ or hypothesizing the reasons for these variations.

Most outbreaks in NHPs were recorded in urban or peri-urban areas (81.9%, *n* = 963/1,175), with Lago Norte, Lago Sul, Candangolândia, and Guará being the RAs with the highest number of records in the FD. Favorable environmental conditions for NHP populations, such as vegetation, shelter, and food availability, can enable the establishment of marmoset populations and facilitate their movement in highly urbanized areas^21^ ^,^ ^22^. Unfortunately, it was not possible to determine the primary reasons for these findings because of the lack of studies on NHP population dynamics in the urban and peri-urban areas of the FD. In addition, the propensity of residents to report NHP deaths and the higher density of populated regions may increase the likelihood of sick or dead animals being found and reported to the GVAZ/SES-FD, possibly explaining the higher number of NHP deaths recorded in certain areas.

As a health surveillance service, the GVAZ/SES-FD demonstrated consistent results, sampling most NHP deaths for pathological and YF diagnoses (96.7%). Unfortunately, a YF diagnosis was not performed in approximately 21% of the sampled carcasses because of advanced decomposition. In studies on the surveillance of deaths among NHP during severe YF outbreaks, most animals could not be sampled or tested for YFV infection^1^ ^,^ ^14^. Poor carcass preservation and difficulties in adequate sampling under field conditions have been identified as the leading causes of improper YF laboratory tests^1^ ^,^ ^3^ ^,^ ^14^. The small territorial extent of the FD, encompassing a large metropolis, such as Brasília, and extensive urban areas, possibly facilitated the reporting of deaths, collection of dead NHPs, and harvesting of samples for YF diagnosis.

The surveillance of NHP outbreaks aimed at preventing YF in FD experienced a significant reduction of approximately 60% in inconclusive diagnoses over the last 5 years (2018-2022) compared to the previous 10 years. Traumatic injuries (61.3%) and infectious diseases (31.2%) accounted for the majority of deaths. Recently, acute toxoplasmosis and electrocution were identified as some of the most important causes of infectious and traumatic deaths in marmosets in this region^16^ ^,^ ^23^ ^,^ ^24^. Other studies investigating the causes of death in NHPs in Pernambuco and Rio Grande do Sul have reported conclusive diagnostic rates ranging from approximately 53% to over 68%, with traumatic injuries and infectious diseases being the most frequent diagnoses^25^ ^,^ ^26^. In September 2017, as part of the Brazilian Ministry of Health's program for the decentralization and expansion of its Official Laboratory Network, the Regional Laboratory for the Diagnosis of Outbreaks and YF in NHPs was established at the University of Brasília, Brazil. This initiative resulted in the systematization of necropsies for all collected dead animals, as well as sampling for YF, thereby reducing the rate of inconclusive FD diagnoses.

This study also identified some challenges in the surveillance of NHP outbreaks in FD. Despite the significant increase in sampled animals over the last 5 years, following a peak in 2018, there was a marked reduction in the number of investigations into NHP deaths in subsequent years. Additionally, almost half of the animals had no recorded locations of NHP death, a considerable number of carcasses could not be sampled because of decomposition, and many animals had inconclusive pathological results despite testing negative for YF. These findings suggest a potential decline in NHP outbreak surveillance, which may be attributed to factors such as decreased public sensitivity to reporting animal deaths, a possible decline in NHP populations, the weakening of local health surveillance services, and other undetected causes.

## CONCLUSIONS

Systematic surveillance of NHP outbreaks in FD has underscored its strategic importance and unique characteristics, mainly due to the predominance of marmosets and the low positivity rate for YF in the region. Given its strategic location in Central Brazil, the strengths and challenges of YF surveillance in FD demonstrate the need to improve and enhance the surveillance system. Such improvements are essential for better anticipation and development of strategies for YF prevention and population protection.

## Data Availability

Research data is included within this article.
